# Exploring Protective Alleles in the Pathogenesis of Alzheimer’s Disease Using Mouse Models

**DOI:** 10.1002/alz.087013

**Published:** 2025-01-03

**Authors:** Kim N. Green

**Affiliations:** ^1^ Institute for Memory Impairments and Neurological Disorders (MIND), Irvine, CA USA

## Abstract

**Background:**

Several variants have been identified that protect against the development of Alzheimer’s disease (AD). Understanding how these alleles convey protection inform us not only about the disease pathogenesis, but also guide therapeutic strategies. The UCI MODEL‐AD consortium has developed several protective alleles including a putative gain of function variant of ABCA7, and the APOE Christchurch variant. Here we have explored the impacts on these variants in animal models of AD to elucidate their mechanisms of protection.

**Method:**

CRISPR‐Cas9 was used to introduce either murine equivalent of ABCA7 V1613M, or APOE R136S (Christchurch) into the murine genome. Mice were then bred to homozygosity with either the 5xFAD mouse model of amyloidosis (Abca7 and Apoe), or the PS19 mouse model of tauopathy (Apoe), and then phenotyped across multiple ages using histology, biochemistry, and single cell spatial transcriptomics and proteomics.

**Result:**

*Abca7* ^V1613M^ variant dramatically reduced Ab production, protected against the formation of parenchymal plaques, and against increases in cortical and plasma neurofilament light chain. *Apoe* ^R136S^ produced divergent effects in glial populations depending on the stimulus. In 5xFAD mice it decreased plaque number and size, and increased the microglial response to the plaques, as evidenced by upregulated disease associated gene expression and protein expression. In PS19 mice tau pathology was mildly increased, but the glia response was markedly suppressed with *Apoe* ^R136S^. Plasma neurofilament light chain was reduced by *Apoe* ^R136S^ in 5xFAD mice but not PS19 mice.

**Conclusion:**

We highlight that the primary mechanism of ABCA7 appears to be via the production of Ab and plaques. On the other hand, APOE Christchurch dramatically effects glial responses to pathology, notably increasing microglial reactivity to plaques, but markedly suppressing their response to tauopathy.

